# Sexualized relations in the workplace: the end of a taboo? A perspective on-state-of-the-art research and issues of regulation by work institutions

**DOI:** 10.3389/fsoc.2026.1799827

**Published:** 2026-05-19

**Authors:** Stéphanie Monay, Morgane Kuehni

**Affiliations:** School of Social Work, University of Applied Sciences and Arts Western Switzerland (HETSL | HES-SO), Lausanne, Switzerland

**Keywords:** consent, gender, regulation, sexual harassment, sexuality, sociology < discipline, work

## Abstract

Sexualized relations in the workplace have received limited attention beyond research on sexual harassment. This article reviews legal, organizational/managerial and sociological literature on so-called “consensual” sexualized relations, bringing together the scarce studies written in French with a more prolific scientific literature written in English. It identifies key issues in these three disciplinary fields, as well as the terminology used to define and analyze such relations. By mobilizing the concept of a continuum of sexualized relations at work, it highlights underexplored questions for the sociology of work and gender studies. The concept of a continuum not only enables a critical deconstruction of the binary categories opposing consent to harassment, but more fundamentally it renews the analytical framework by conceptualizing sexualized relations in the workplace as a structural phenomenon, shaped by work organization and conditions, as well as by the norms and power relations that govern professional environments. In conclusion, this concept makes the regulation of sexualized relations at work a fully-fledged research question: in a post-#MeToo context, it makes it possible to ask not only who is protected by regulatory mechanisms, but whether those mechanisms reproduce or reconfigure the very power relations they claim to address.

## 1 Introduction

Sexualized relations in the workplace—understood as interactions socially constructed as sexual (Bozon, 2001a) that occur in or arise from working relationships—constitute a blind spot in the scientific literature. Existing research has approached them predominantly through the lens of sexual harassment, thereby narrowing the analytical scope of workplace sexuality: while this body of work has been productive, it has also tended to frame sexualized interactions primarily in terms of harm, foregrounding a binary opposition between consent and coercion. This binary, however, risks obscuring the power relations that structure all sexualized interactions at work, including those commonly described as consensual. Since the 1970s, sexual harassment has been a major driver of research in this field, with a marked acceleration following the #MeToo movement (Saguy and Rees, 2021; Schultz, 2018). The studies focus on the harmful consequences of sexual harassment for workers, particularly women (Mackinnon, 1979; Mehta, 2023). In this context, there are debates between those who advocate for the “desexualisation” of workplace (Dworkin, 2007; Mackinnon, 1979) and those who view this trend as an intellectual impasse (Avril, 2019; Williams et al., 1999). At the same time, knowledge about sexualized relations at work remains limited, especially with regard to relationships framed as consensual. This gap reflects not only the persistence of taboos surrounding sexuality—particularly in professional settings (Lapeyre, 2018)—but also the inherent difficulty of characterizing what is or is not “sexual” (Bozon, 2001a). As a result, the analytical tension between harassment, consent and power relations remains insufficiently explored, even though it is precisely this tension that structures the full spectrum of sexualized relations in the workplace.

This article aims to give an overview of the state of knowledge and scientific research done in the francophone^1^ and anglophone worlds on sexualized relations in the workplace, including “consensual” relationships between colleagues. It falls into three parts. The first part outlines the main results found in the literature on sexualized relations in the workplace in three disciplinary fields: the law, organizational and management studies,^2^ and sociology. This overview allows us to identify how sexualized relations at work are addressed in those three fields: how are they dealt with in research? A shared blind spot emerges: sexualized relations that fall outside the category of sexual harassment remain largely under-theorized. Legal frameworks address them only in exceptional cases, organizational and management studies reduce them to questions of performance and risk, and sociological research, though more attentive to power dynamics, has yet to consolidate them into a coherent analytical object. The second part circles back to terminological issues: how are sexualized relations named and defined by researchers? Among the various suggestions put forward, we chose the concept of a “continuum of sexualized relations at work” proposed by Avril (2019). This concept sheds light on the wide range of possible sexualized behaviors and interactions in the workplace and supports the idea that all of them, not just sexual harassment, contribute to the (re)production of social and gendered relations of power. Based on this continuum, the third part of the article summarizes what was identified in the literature as the most important effects that sexualized relations in the workplace have on institutions, work collectives and individuals. It highlights how such consequences cannot be deduced simply from isolated variables (forms, motivations, individual characteristics, etc.) but are socially constructed from the basis of a complex web of institutional, professional, and social norms, which are highly dependent on the organizational logic specific to work institutions. Finally, the conclusion explores the key issues associated with the regulation of sexualized relations by work organizations, and how the concept of a continuum can be useful in analyzing them. Although regulation is a key element in understanding the experiences stemming from sexualized relations at work, it still fuels precious little scientific investigation.

## 2 Sexualized relations in the workplace: what does the literature say?

Sexualized relations at work have long been overlooked^3^ because they challenge a deeply-rooted social construct: sexuality belongs to the private sphere, to leisure and pleasure, unlike work, defined by constraint, subordination and the effort required to produce results (Hearn et al., 1989). However, research shows that sexualized relations are widespread, affecting between 22% and 75% of individuals, depending on the source [see in particular: SHRM (Society for Human Resource Management), 2023]. As the measures used to quantify the phenomenon encompass very heterogeneous realities, it is difficult to identify trends. However, the literature suggests an increase in such relations (Doll, 2024). While the phenomenon is of great interest to the tabloids—especially when it involves executives, such as the recent case of the CEO and human resources director of the US start-up *Astronomer* or the CEO of the multinational *Nestlé* who was fired for having a relationship with a subordinate—it remains understudied. This section reviews how sexualized relations at work are addressed in three disciplinary fields, updating the reflections of Williams et al. (1999).

This article provides an interpretive synthesis of the available knowledge. The review process is structured in successive cycles and it examines the research questions raised, the theoretical frameworks employed, the empirical findings produced, as well as the dominant perspectives and blind spots in the literature. It was conducted in several phases and covers three disciplinary fields, presented here in summary form: law, organizational and management studies, and sociology. The initial phase of the research, conducted from November 2023 to April 2024, combined keywords and query searches^4^ in French as well as British and American English, with the objective of identifying references linking sexuality and work. Searches were conducted on *Web of Science, Google Scholar, Scopus, Cairn.info* and *Persée*, spanning a broad period from the 1970s to the present and incorporating both seminal works and the most recent developments. This phase identified more than 200 references. These initial searches revealed a notable prevalence of work in organizational and management studies, and a focus on sexual harassment. Reviewing these early studies enabled us to identify the concepts employed and to refine the keywords used, which vary significantly across research fields (see Section 3). The second phase of the literature review introduced additional keywords for verification purposes, as well as an examination of the references cited in the identified publications (“snowballing” approach). The selection process was informed by the objective of expanding the scope beyond sexual harassment and of promoting disciplinary diversification to mitigate the predominance of organizational and management studies. The search was systematically expanded through October 2025 with the aim of focusing on works that explicitly address sexualized relations in the workplace within the context of the organizational and professional conditions in which they occur. At the end of this process, a corpus of approximately 250 references (scientific articles, book chapters and books) was compiled.

The present article draws on about 100 references deemed most relevant to the subject matter and objectives: on the one hand, to offer a structured and critical analysis of current knowledge; and on the other, to identify existing gaps and lay the groundwork for an analytical framework centered on the continuum of sexualized relations at work and capable of guiding future research. This approach thus aims to be both cumulative and heuristic, contributing to the construction of a theoretical proposal informed by the state of the art.

### 2.1 Legislating on sexualized relations at work: what gives the right to do so? Legal approaches

Since the 1970s, debates have revolved around sexist and sexual violence in the workplace (Dworkin, 2007; Mackinnon, 1979, 1997) and have focused mainly on sexual harassment, both in the United States (Hallinan, 1992; Marshall, 2005; Schultz, 2018) or in France (Lieber et al., 2019; Saguy, 2003, 2012). While the fight against sexual harassment in the workplace now has a legal basis in most Western countries, the question remains as to how much leeway employers have to prevent sexual harassment: is it legally possible to prevent or even prohibit the relations often referred to in the literature as “workplace romances”?

There is no clear-cut answer to this question. The literature stresses the importance of national legal culture, particularly the balance between the right to privacy of individuals and the right that companies have to ensure proper functioning, as well as the employer's duty to prevent and punish sexual harassment in the workplace (Boyd, 2010; Géniaut and Mathieu, 2023; Hallinan, 1992). Depending on the country concerned, this balance tips either toward the primacy of individual rights, as is the case in most European countries, in Japan, China, and most South American countries, or toward organizational rights, like in the United States, Canada, and Australia (Fiorentino, 2020; Labatut, 2023).

In general, labor law is not concerned with consensual relationships (Géniaut, 2023), and does not dictate prescriptive or prohibitive standards, except in cases of sexual harassment. However, analyzing case laws in certain countries shows that sexualized relations at work have caused disputes between workers and employers, mainly in cases leading to layoffs (Fiorentino, 2020). In France, the prevailing approach is to weigh up interests on a case-by-case basis, where “the protection of the employee's privacy prevents any retaliation by the employer (with some exceptions).” (Fiorentino, 2020, p. 9). This protection is not as strong in the United States, where work institutions are allowed to implement restrictive internal measures and even impose punitive sanctions which may reach the point of dismissal (Boyd, 2010; Wilson et al., 2003).

### 2.2 Love and/or performance. Approaches in organizational and management studies

Organizational and managerial literature initially focused on desexualization mechanisms in work organizations (Burrell, 1984) and the role of sexuality in the construction of organizations, and *vice versa* (Hearn et al., 1989; Hearn and Parkin, 1987). More recent work examines “organizational romance” mainly from the perspective of business performance and productivity (Horan and Chory, 2009; Rabeau, 2020). Literature reviews conducted at different times (Anand et al., 2023; Panda and Puri, 2025; Pierce et al., 1996)^5^ show how little the issues raised have changed since the 1970s.^6^ Two main areas of consensus emerge. First, the relationship characteristics (hierarchical or horizontal, (extra)marital, perceived motives for the relationship) are central to how it will be received and regulated by work collectives and organizations (Cowan and Horan, 2014). Secondly, the impacts of sexualized relations on the work environment and on professional opportunities are gendered: women face more negative consequences (Chory and Hoke, 2019; Lickey et al., 2009).

This literature identifies the “consequences” of sexualized relations on individuals, work collectives and the organization, in order to determine whether they are beneficial or detrimental to performance. It has mainly focused on the effects of “workplace romances” on the company (Horan and Chory, 2009; Rabeau, 2020), noting that they have (predominantly) negative and (secondarily) positive consequences on the performance and productivity of workers (Anand et al., 2023). However, some studies tackle the effects of those “romances” on workers, particularly their impact on their mental and physical health (which include lack of commitment, lesser psychological wellbeing, greater physical tension, etc.; Berdahl and Aquino, 2009). Those studies primarily examine the effects of sexual harassment (Mehta, 2023), but also a “potential for deterioration” associated with consensual relationships, such as the proliferation of rumors and gossip in the workplace or breakups (Chory and Hoke, 2019). Consensual sexualized relations are also identified as potential factors for wellbeing at work (Mehta, 2023; Pierce et al., 1996): they cause less stress, anxiety, frustration and likelihood of burnout; more commitment and job satisfaction, more pleasure, energy and self-esteem (Aquino et al., 2014; Gillanders et al., 2021; Mehta, 2023). Overall, these contributions highlight the ambivalent effects of sexualized relations on both individual wellbeing and organizational performance, with a clear emphasis on the latter.

Research also examines the organizational measures implemented to regulate sexualized relationships in the workplace. Largely produced in the United States (Panda and Puri, 2025), it supports organizations' right to regulate such relationships by means of risk minimization, which focuses on the costs incurred by organizations, particularly in terms of productivity (Kolesnikova and Analoui, 2013; Mainiero, 2020; Pierce and Aguinis, 2009). The organizational measures identified in the literature include punitive sanctions (reprimands, layoffs), administrative control and surveillance (love contracts, removal from supervisory roles), but also “positive” management decisions or even the encouragement of sexualized relations at work (counseling, a labor organization favorable to couples; Saint-Onge and Ritory, 2015). While the United States tend to place sexualized relations in the workplace under contract, there is consensus in the literature about the fact that few organizations have implemented formal measures so far. Dealing with “workplace romances” is rather a matter of managerial discretion and informal mechanisms (Riach and Wilson, 2007). Nevertheless, recent publications advocate proactive measures as regards those issues in order to prevent sexual harassment in the workplace, which is detrimental to individuals and to the image of the organization (Anand et al., 2023; Cavico and Mujtaba, 2021). Although the measures to be taken in the case of lateral relations (without a hierarchical relation) are still being debated, there prevails great consensus on the subject of hierarchical relations, which are less tolerated and more adamantly prohibited (Lickey et al., 2009). Finally, the consequences of organizational policies and practices for managing sexualized relations *on individuals* remain underexplored (Saint-Onge and Ritory, 2015).

### 2.3 Sexuality: a tool for male domination at work. Sociological approaches

Sexualized relations remain the “black box of the sociology of work,” (Deruelle, 2022, p. 90), even though studies have long highlighted their importance in factories (Aumont, 1953; Roy, 1974). While there is a core of sociological research in English-speaking countries (Adkins, 1991; Cockburn, 1991; Dellinger and Williams, 2002; Gutek, 1985; Pringle, 1989; Williams et al., 1999), in France, research on sexuality at work is loosely connected to sociology of work (Oeser, 2019), and the available information is most often fragmented within ethnographies of professional environments.

An entire field of the sociological literature addresses sexualized relations between professionals and clients or patients. This is the case in several studies drawing on emotional labor (Hochschild, 2012) in highly feminized professions such as hospitality and catering, sales, health or social work (Green, 2023; Kensbock et al., 2015; Kuehni and Bovey, 2018; Molinier, 2011). This research shows that the emotional labor of managing and regulating sexuality at work is rendered invisible and falls upon women and dominated groups (Martin et al., 2014). These situations arise not only from the type of activities performed, but also from organizational forms: professional norms, labor division, gender diversity, hierarchical and collaborative structures, etc. (Boni-Le Goff, 2024).

A second strand addresses sexualized relations between colleagues, particularly in professions with low gender diversity. In male strongholds, where virility is valued through sexual performance (Pruvost, 2008), women are highly sexualized, systematically face sexism and often have to fend off colleagues' advances (Boni-Le Goff, 2016; Gruber and Morgan, 2005; Guichard-Claudic and Kergoat, 2007; Kuehni et al., 2018). They bear the responsibility of keeping sexuality out of work relationships (Lapeyre, 2018; Monay, 2018; Rey et al., 2022). When men enter female-dominated fields, sexualized relations with female colleagues or clients help restore their masculinity and/or avoid stigma associated with being a man in a “women's job” (Buscatto and Fusulier, 2013). It is also a way to dispel suspicions of homosexuality (Cross and Bagilhole, 2002; Olivier, 2015).

A third strand focuses on effects of sexualized relations on individuals' careers. It centers on highly competitive professions such as politics (Bargel et al., 2013; Jérome, 2014) or academia (Bellas and Gossett, 2001; Cardi et al., 2005; Vohlídalová, 2017). Sexualized relations are ambivalent and represent resources and dangers for careers, but above all they exemplify a “factor of professional inequality” (Deruelle, 2022, p. 89) between women and men, keeping women away from prestigious positions. Similar findings emerge in artistic professions, which are confined worlds based on interrelations, where relationships and reputation are important for one's career (Buscatto, 2015) and where “sexuality, seduction, and all forms of self-presentation function as means of acquiring social capital and therefore as elements of recognition” or lead to exclusion (Bozon, 2001b, p. 16). Artistic fields are marked by sexualization produced through professional socializations (Hubbs, 2004; Sorignet, 2004), especially when the body is central to the work done (Doyon and Katuszewski, 2017). This sexualization can normalize sexual violence against women (Trachman, 2018), especially in elite cultures (Buscatto et al., 2021). These fields—politics, academia, arts—also share the central role of teaching or mentoring relationship “in which hierarchical relations can be eroticized, contributing to a blurring of the lines between learning, seduction and sexual harassment.” (Trachman, 2018, p. 132). These studies show that “sexualization of professional relations is imposed by men” (Boni-Le Goff, 2016, p. 166), and negatively affects women's careers (Cardi et al., 2005; Delvaux et al., 2015).

Numerous studies highlight gendered violence inherent in sexualized relations at work (Clair, 2013; Fassin, 2009; Williams et al., 1999), showing that they constitute a form of arduousness for women's work, which leads to physically and psychologically exhausting situations (Lapeyre, 2018). Nevertheless, some ethnographies show that so-called consensual sexualized relations can act as “remedies” to work hardship, used as “ways to take off pressure” (Pruvost, 2007) or “safety valves” (Monjaret, 2001) in difficult and stressful work conditions (Pringle, 1989). This supports the relevance of analyzing sexuality not only as “risky behavior” at work, but also as “an element of individual wellbeing” (Bozon, 2001a, quoting Gall, 2001).

A major contribution of sociological literature is to emphasize the socially constructed nature of sexualized relations at work, showing that they are interpreted differently depending on individuals. Dellinger and Williams (2002), Giuffre and Williams (1994) and Williams et al. (1999) show that labeling sexual harassment in the workplace is not always straightforward and depends on social background, professional and organizational norms, and national social and legal contexts. This is supported by the analyses made in France by Cartier and Retière (2008), or more recently by the special issue on gender and the working-class on the job (Avril et al., 2019), which show that practices perceived as “normal” or “abusive” coexist in the workplace. Those studies emphasize that the perspective on sexualized relations depends on the professional context and varies according to the professional and social categories of individuals and work groups. For example, sexual harassment is less frequently reported in highly sexualized organizational cultures, such as the service sector (Adkins, 1991): sexualized relations are legitimized there by professional norms (Coffey et al., 2023). Workplace climate and equality policies also influence recognition of sexual harassment (Timmerman and Bajema, 2000). Labeling processes depend on power relations among individuals, themselves influenced by organizational and professional cultures, as well as by the social context (sexism, heteronormativity). Other factors include job position (direct and indirect hierarchical relationship, stable or precarious job, level of education, etc.), gender, class, race, sexual orientation, etc. Giuffre and Williams (1994) highlight the existence of a system of “double standards,” i.e., rules and norms that differ according to gender (and race, class, sexual orientation, etc.): identical behaviors are perceived and judged differently depending on the social specificities of the people who behave in a sexual manner and those who are the targets of such behavior. They raise the hypothesis that “identifying behaviors that occur only in counterhegemonic contexts [deviating from the prevailing norms] as sexual harassment can potentially obscure and legitimize more insidious forms of domination and exploitation.” (Giuffre and Williams (1994), p. 380). Their work underlines the importance of framing and ambiguity in the study of sexualized relations at work.

## 3 What conceptual tools may be used to understand and analyze sexualized relations at work?

The very varied terminology used in the three disciplinary fields reflects the fact that this research area is barely mapped out and is subjected to different approaches. In this part, we go back to the various concepts resorted to in the different fields and argue for the use of the concept of “continuum of sexualized relations at work” (Avril, 2019) as developed by sociology.

### 3.1 Love, romance, or sexuality: choosing and using a terminology

When it comes to sexual violence, the terminology is more consolidated across the three disciplinary fields and emphasizes its coercive nature: workplace sexual harassment, sexual violence at work or unwanted sexual attention. The terminology surrounding sexual harassment has developed—through its juridification—in order to clarify its different forms and mechanisms, such as *quid pro quo*, environmental, verbal or non-verbal, etc. harassment.

On the other hand, when it comes to “consensual” relationships, the terms used are manifold and differ across the three disciplinary fields investigated. The law resorts to catchy terms such as: love at work, romantic relationships at work. Organizational and management studies use a particularly striking terminological variety: authors use the term “romance” (workplace romance, romance at work, organizational romance) alongside related vocabulary that uses the term “love” and the adjective “romantic.” The emotional dimension and the allusion to feelings prevail, while a less literature focuses on sexuality and sexual practices (sexuality in the workplace, sexual relationships). The use of the term “romance” reflects a fear of using sexually explicit terminology, but also a desire to separate this phenomenon from that of sexual harassment. However, by referring to feelings intended to be more “noble,” the term “romance” emphasizes a dimension that is removed from sexual practices and that invisibilizes the power relations framing sexualized relations, including the “consensual” ones. Few studies in this discipline take into account the social interactions between the workers concerned and the power relations that are central to sociological literature.

Some studies in the field of organizational and management studies literature lay stress on the uses of sexualized relations by workers. Their perspective affords an interesting terminology, notably the concept of social-sexual behaviors at work. The definitions of social-sexual behaviors at work found in the literature are varied: some authors refer to them as any non-professional behavior with a sexual component in the workplace (Bowes-Sperry and Powell, 1999; Gutek et al., 1990), while others define them in opposition to sexual harassment (Aquino et al., 2014; Berdahl and Aquino, 2009; Gillanders et al., 2021; Mehta, 2023), emphasizing that the parties involved do not perceive those behaviors as coercive, threatening, or harassing. The term “social-sexual behaviors at work” is used in studies highlighting the “positive” impacts of sexualized relations at work on the wellbeing of workers (Mehta, 2023), thus revealing the divergent nature of sexual harassment in its effects. The studies conducted by Aquino et al. (2014) underline the fact that sexualized relations at work take many forms that are not inherently good or bad, but rather depend on social norms^7^ in specific situations, thus revealing the existence of power relations. Individual subjectivity is therefore central to understanding social-sexual behaviors at work, which may be perceived as sexual harassment by third parties, indicating that not only are power relations at play between the individuals involved, but also at the level of the work collective.

However, the authors exclude certain sexualized interactions from their definition of social-sexual behaviors at work and consider them external to the work environment (they see them as belonging to the private sphere) and/or too focused on sexual consumption or procreation to be considered as social practices relevant to work relations (Aquino et al., 2014). Thus, marriage or sexual intercourse between colleagues are excluded, or even “romantic relationships” according to an unsatisfactory dichotomy established between sexuality and feelings. Thinking in terms of the “limits” of work is hardly satisfactory in our opinion, given the growing intricacy of private life with professional life (Berrebi-Hoffmann, 2009), particularly in terms of the impact that sexualized relations at work have on private life (Deruelle, 2024; Green, 2023). In addition, employers' legal responsibility in cases of sexual harassment has gradually extended into areas of interaction on the margins of the professional sphere (such as outings with colleagues, company parties) and all is reinforced by the use of social networks—both professional and non-professional—in sexualized relations at work, which is now commonplace (Cowan and Horan, 2021; Mainiero and Jones, 2013).

### 3.2 A Sociological proposal: the continuum of sexualized relations at work

Sociological literature offers an interesting take to analyze sexualized relations at work and avoid the pitfalls mentioned above while drawing on thought-provoking aspects revealed by different disciplines: that of the “continuum of sexualized relations at work.” (Avril, 2019). Following the logic of Tabet's (2004) concept of the “continuum of sexual-economic exchange” and Kelly's (1987) concept of the “continuum of sexual violence,” the notion of the continuum of sexualized relations at work allows us to address both sexual harassment and “consensual” relations, while revealing the porosity of the boundaries separating different forms of sexualized relations. Indeed, the advantage found in the notion of a continuum is that it does not posit a clear gap between harassment and consent: the binary nature of the two has been empirically discredited (Deruelle, 2024; Williams et al., 1999). Because it is based on “an analytical outline of interpretation,” (Gayet-Viaud, 2021, p. 60), the concept of a continuum of sexualized relations at work offers a comprehensive and situated analysis of these relations and avoids proposing a ranking system classifying them according to a degree of severity, without considering them as equal either (Kelly, 1987).

Mobilizing the concept of a continuum of sexualized relations at work affords several advantages. First, it allows us to regroup all the practices that may be classified as belonging to the realm of “sexuality”—such as flirting, coming on to someone, physical contact, sexual intercourse, etc.—as well as those belonging to the sphere of emotional relationships (Oeser, 2019) that form the background of those practices, such as “one-night stands,” platonic seduction, informal or official couples, extramarital affairs and even marriage. The concept of a continuum also makes it possible to avoid isolating the issue of harassment from other “consensual” sexualized practices, emphasizing the porosity of boundaries, without minimizing the power dynamics characteristic in situations of sexual harassment (Williams et al., 1999). Fassin (2012, p. 57) calls for breaking away from the idea of consent “defined in terms of pure will” and considers that the choices and decisions of people are never completely free from social norms and power relations. As Bozon (2001a, p. 7) writes, “as of today, sexual negotiation is not conducted between equals,” because systemic inequalities remain.These clarifications on the notion of consent allow us to return to the conceptual break between power relations and the relations of domination developed by Foucault (2001, p. 1056): power relations, however restrictive they may be, leave open “a field of possibilities where several behaviors, several reactions, and various modes of conduct may unfold. Where determinations are saturated, there is no power relation,” but rather violence and domination. For Fassin (2012, p. 59), it is precisely this “tension between freedom and power that marks the irreducibility of the latter to domination” and that state of things defines the complexity of social relations. Therefore, the idea of a continuum allows us to refer to a set of relations and practices: although they are always determined by power relations, it is only in certain situations that violence and domination prevail. Thinking in terms of a continuum thus makes it possible to “shift the question of consent to consider, not the individual as free or coerced, but the subject (in Foucault's sense) who emerges from a power relation.” (Fassin, 2012, p. 66). This opens up perspectives to think through the agency of subjects, which has been highlighted in sociological work (Lerum, 2004; Loe, 2007; Williams and Dellinger, 2010; Yelvington, 1996), as well as the possibility of thinking changes or reconfigurations in power relations (Legouge, 2012). The notion of a continuum therefore allows us to pay particular attention to “the confusion that obtains in people's minds” and to the “capacity for resistance” of individuals which is characterized in particular by the organizational and social conditions prevailing in work institutions (Cartier and Retière, 2008, pp. 81–82).

We propose to define sexualized relations at work like all sexualized interactions—as socially constructed as sexual—taking place within the world of work or stemming from work relations. These relations involve different individuals from the immediate or distant work collective (colleagues, superiors, subordinates, interns, suppliers, consultants, etc.), or even a person who belongs to the clientele, the patient base, or the student body of the workplace. The use of the adjective “sexualized” is central to this definition, as it highlights the process of sexualizing behavior that is not inherently sexual but socially constructed as such (Bozon, 2001a): the practices and meanings associated with them are deeply rooted in the social norms and practices that shape the everyday spaces of daily life (Bozon, 2001b, 2018)—including the workplace. Sexual harassment and sexual violence in the workplace are sexualized relations set at the extreme end of the continuum along which relations of domination manifest themselves, i.e., at the point where the freedom of choice and the freedom of resistance of the individuals involved are crushed. Other forms of sexualized relations (referred to by organizational and management studies literature as social-sexual behaviors at work) imply forms of sexualized relations whose social uses and effects cannot be defined normatively *a priori*, as those depend on the power relations that come into play in specific situations and interactions. In those relations, agency, as “freedom” or room for maneuver, is evident, as sexuality can be used both as a means of exercising power and as a means of resisting it (Oeser, 2019), and this also holds sway for women. Yelvington (1996, p. 318) has shown how black and Indian women workers in Caribbean factories use flirting “as part of tactics of resistance and power-seeking.” Sexualized relations at work thus serve as a means of empowerment, showing that socially-dominated groups should not be considered as merely passive entities, but as subjects (Loe, 2007; Yelvington, 1996).

Our critical review of the literature in the fields of law, organizational and management studies and sociology highlights the conceptual distinction between social-sexual behaviors and sexual violence, while underlining the practical ambiguity of perceptions and qualifications in concrete social interactions. The intersection of the three disciplinary fields reveals the difficulty of analyzing sexualized relations at work, due to their being so tightly intertwined with different systems of systemic, institutional, professional and individual norms. Attempting to define sexualized relations at work on the basis of their forms, i.e., elements that are supposed to be “objective” such as behavior (e.g. touching the buttocks, ogling, engaging in sexual intercourse) or the organizational, professional, or social characteristics of the individuals involved (gender, direct or indirect hierarchical relations, etc.) would be to absolutely no avail. Those elements must indeed be taken into account (see Table 1), but they do not suffice because the sexualization of relations is a social construct inherent to social experiences and perceptions, as well as to organizational and professional norms. This complexity is particularly well demonstrated in the scientific literature on sexual harassment: while its classification can now be based on legal definitions, legal debates and case law,^8^ it remains the subject of an ongoing struggle, particularly among feminist groups, which emphasize that the production of the norm (Le Magueresse, 2014) is not a process that can reach a once-and-for-all conclusion (Saguy, 2003, 2012; Schultz, 2018). Sexual harassment, like all sexualized relations in the workplace, must be understood as a social construct or a process that cannot be defined in fixed and rigid terms (Weeks et al., 1986). The central issue therefore lies in identifying the social practices and mechanisms through which power relations become relations of domination, even when individuals are not aware of them (Dellinger and Williams, 2002; Giuffre and Williams, 1994).

**Table 1:** Continuum of sexualized relations at work and their consequences.

Power relations		Relations of domination
Consensual and ambiguous social-sexual behaviors		Sexual violence
Identified forms of sexualized relations at work		
**Legally authorized** • Flirting (eye contact games, jokes, banter, etc.) • Coming on to someone • Physical contact • Casual or regular sexual intercourse • Official/advertized romantic/sexual relations • Institutionalized forms (marriage, partnership, cohabitation, etc.) • Affaires • Breakups	**Organizational positions** • Lateral/horizontal relationship • Direct or indirect hierarchical relations • Pedagogical or mentoring relationship • With patrons, patients, users, etc.	**Subject to conviction** • Violence, rapes and sexual aggressions • Q*uid pro quo* sexual harassment • Direct sexual harassment • Hostile work environment sexual harassment • Verbal/non-verbal harassment
Identified consequences for individuals, work collectives and work institutions		
**Positive, ambiguous or negative impacts on** • Physical and mental health of individuals • Feeling of wellbeing and job satisfaction of individuals • Professional career and reputation of individuals • Working conditions of individuals and work collectives • Performance/productivity of individuals, work collectives and organizations • Relations between individuals, work collectives and organizations • Organizational image and reputation		**Negative impacts on** • Physical and mental health of individuals • Feeling of wellbeing and job satisfaction of individuals • Professional career and reputation of individuals • Working conditions of individuals and work collectives • Performance/productivity of individuals, work collectives and organizations • Relations between individuals, work collectives and organizations • Organizational image and reputation

## 4 Discussion. For a contextualized and integrated analysis of sexualized relations at work: the central role of professional and organizational norms

### 4.1 Consequences of sexualized relations in the workplace

The literature examined identifies the consequences resulting from sexualized relations in the workplace on three levels: on individuals, on work groups and on work institutions (see Table 1). While research clearly highlights the negative consequences of sexual harassment on those three levels, the results are less consistent when it comes to other forms of sexualized relations. The impacts on health, job satisfaction, career and even institutional image are varied. Far from any mechanical logic, the scholars working on the subject reveal, for example, that certain sexualized relations at work that meet all the normative criteria of a “healthy and happy” relationship between two individuals can have negative consequences in the workplace (Avril, 2019; Bellas and Gossett, 2001). The “professional advantages and disadvantages” (Deruelle, 2024, p. 17) associated with sexualized relations at work, which may go on throughout a person's career, depend more on the reaction of work collectives and organizational and professional norms than on the characteristics of the relation itself (Srinivasan, 2022; Williams et al., 1999).

### 4.2 Power dynamics and organizational characteristics

Sociological literature shows that the specific organizational characteristics of each workplace give rise to varying approaches and practices for managing sexualized relations at work, with diverse consequences for different social and professional groups (Giuffre and Williams, 1994). A comparative reading of this literature, across different fields and professional sectors, shows that the forms and consequences that sexualized relations have depend on the power dynamics established by organizational characteristics, such as the degree of social diversity, the hierarchical rigidity, the professional cultural norms of masculinity or femininity, or the organization of work (schedules, promiscuity, intensity, etc.).

Thus, any analysis of the “social organization of sexuality” (Laumann et al., 2000) in the workplace must consider the structuring role of organizational and professional norms and configurations. The contrasting cases of the restaurant industry and of the military provide a good illustration of the different dynamics regulating sexuality depending on organizational norms, with various consequences affecting workers. Those two empirical examples, conducted prior to the “#MeToo movement” (Pavard et al., 2020), allow us to grasp the importance of analyzing the mechanisms produced by the organizational regulation of sexuality in the workplace. Research by Giuffre and Williams (1994) in the restaurant industry has shown that the high sexualization of tasks, constant emotional labor and the normalization of sexual exploitation for commercial purposes (to attract customers, get better tips) lead to the management of sexuality by individuals and the work collective (Loe, 2007). In this professional context, “individual employees are often forced to draw the line for themselves to distinguish legitimate and illegitimate expressions of sexuality,” (Giuffre and Williams, 1994, p. 381) making it all the more difficult to label sexual harassment as such in their daily work. The authors clearly show how the process of labeling, which is the responsibility of workers, is based on differences in professional status and social differences in race and sexual orientation. In this context, the sexualized behavior of people at the bottom of the professional hierarchy is more easily considered harassment. The behavior of Mexican cooks, who combine a subordinate professional position with racial and cultural otherness *vis-à-vis* the predominantly white service staff, is thus more likely to be identified and denounced as sexual harassment, reinforcing the precariousness of their employment. This is also the case for gay workers *vis-à-vis* their heterosexual male colleagues, since the sexualized behavior of the former is considered a threat to the hegemonic masculinity of the latter.

Monay's research (Monay, 2018, 2024) on the military, which comes across as an unequal gender regime where a virile and high-performing hegemonic masculinity is promoted, reveals another reality. In this professional context, sexuality at work, assessed as a “problem,” is regulated by explicit but unofficial institutional norms that have consequences on women's daily life in the military. Women, who make up a very small minority of the workforce (less than 2%), are perceived both as intruders in this male-dominated and heteronormative world, as well as organizational “difficulties” because they “bring sexuality into the barracks.” Defined from the outset as variables in the management of sexuality *intra muros*, female military personnel must exercise (self-)control over their bodies and femininity, containing their own desire and that which they might spark in their male colleagues, in order to protect their own reputation as well as that of their company. “Consensual” sexual relations with a fellow soldier are not uncommon due to the great and protracted proximity existing at work but they often remain concealed since they are sometimes prohibited by local hierarchies, or revealed once the relationship is deemed sufficiently “serious.” Those situations do not prevent the reactivation of gendered sexual stereotypes targeting women: couples with a hierarchical difference—women with men of higher rank—cast suspicion not on the men who might be taking advantage of their position of power, but on the women who are thought to reap benefits and privileges from the situation, and are thus discredited professionally. In addition, logistical arrangements are put in place to protect the military and its male officers from “false accusations” of sexual assault or harassment, such as requiring the presence of two officers during promotion interviews for women, which is not the case for men.

These two examples show that both the sexualization and the desexualization of workplaces have negative effects on dominated social groups (women, racialized people, subordinates, LGBTIQ+ community), such as the reinforcement of heteronormativity, hypersexualization, or exclusion (Williams et al., 2009), while protecting the dominant ones. While the literature on sexualized relations in the workplace involving professors, PhD candidates, and students has sometimes explicitly raised the issue of the challenges and consequences of regulating sexuality at the institutional level (Deruelle, 2024; Srinivasan, 2022), this field of inquiry deserves to be broadened beyond mentoring relationships.

## 5 Conclusion. Work organizations and sexualized relations: is regulation meant to protect better or to control better?

The concept of a continuum of sexualized relations at work is a useful tool to make sexuality operational and to “construct [it] as a subject of research [...] [as well as to] study it for what it is: an ordinary dimension—as a social fact—of workplace relations.” (Avril, 2019). It allows us to integrate not only what is at stake between individuals (at the micro level), but also to see the organizational and professional issues specific to different work contexts (at the meso level). It also sheds lights on what is and is not permitted by the legal framework and the weight of local policies and demands related to professional equality as well as the prevention of sexist and sexual violence at work (at the macro level). However, one important dimension of this continuum remains largely unexplored: the power and domination relations linked to the organizational and collective regulation of those relations, which may have negative consequences for the individuals concerned (Avril, 2019). Admittedly, the regulation of sexualized relations by work institutions is an issue at the heart of legal and organizational/management studies. In the face of sexual harassment, the law questions the competition between individual and organizational rights, and organizational and management studies reflect on the integration of this organizational responsibility in the quest for performance, seeking to minimize risks. However, we believe that the issues at stake when it comes to regulation are much broader: who should regulate sexualized relations in the workplace (the organization, the collective, individuals—and if so, what individuals)? What field(s) should it apply to? Who should be protected and for what reasons? How do individuals experience the different forms of regulation? Do regulatory instruments reshuffle power and domination relations or do they, conversely, reproduce them?

Until now, researchers from various disciplines have shown little interest in the consequences of regulation, control and surveillance mechanisms on individuals or work collectives, as most of those mechanisms are very recent. This issue seems all the more important to us as legislative changes on sexual harassment in the workplace increasingly require organizational action. In Europe, contrary to what prevails in the United States, there is little regulation of those relations by labor institutions, particularly through formal means such as love contracts, guidelines extracted from employee or ethics charters. Sociological literature shows that the most widespread mechanisms for controlling and monitoring workers remain peer regulation and individuals themselves. Indeed, sociological studies that have directly addressed sexualized relations in the workplace show how collective or individual regulation depends on the professional culture, and the statutory and social characteristics of the people present in the workplace.

While a few studies have more explicitly explored the “negative” or “positive” effects that the organizational regulation of sexualized relations in the workplace have had—in particular the effects on job satisfaction and productivity (organizational and management literature), and the reconfigurations and reproductions of inequalities and power relations (sociological literature)—we believe it is crucial to develop empirical research on sexualized relations at work and on the organizational, collective, and individual mechanisms regulating them. Fundamentally, the tension between autonomy and protection (Avril et al., 2019) constitutes a central theoretical issue shaping the analysis of sexualized relations in the workplace. On the one hand, regulation aims to protect individuals, particularly those in subordinate positions, from harassment, coercion and structural inequalities. However, it also raises issues related to privacy, freedom and the risks of organizational interference in the private sphere. Instead of pitting these two dimensions against each other, it is important to analyze how they interact in specific organizational contexts, and how regulatory instruments are implemented and adopted. As Srinivasan (2022, pp. 243–250) points out, “top-down” regulation involves the risk of violating individuals' fundamental rights, as the former is designed solely to protect institutions, and it could reinforce control over women and dominated groups. Violence can also stem from the regulation of sexualized relations at work, acting as a “morality police,” determining, for example, who has the right to flirt, have sex, get married, and with whom. In the context of the uneven institutionalization of the #MeToo movement in workplaces (Cavalin et al., 2022; Perrier, 2022), it is also important to examine how regulatory frameworks, whether formal or informal, can serve as resources for individuals and which individuals in particular. Indeed, recent studies highlight a “#MeToo effect” on marginalized groups of workers' awareness of their rights, leading them to use legal and reporting tools (Le Magueresse, 2022). However, this dynamic does not uniformly translate into a real agency because this capacity is unevenly distributed across work contexts, as well as the social and professional characteristics of individuals (Bull, 2025; Lechaux, 2022).

The key challenge is to move beyond a moral issue to a research issue, which is necessarily empirical, in order to question how and why forms of regulation can be experienced as a protective factor or as a risk factor at work (invasion of privacy, control, loss of autonomy, incapacity, hardship, emotional overwork, etc.). The expectations of individuals on this issue differ, depending on whether they are employers—who favor institutional regulation (Boyd, 2010)—or employees—who favor “flexible” and limited regulation targeting sexual harassment (Technologia Universit é Paris 1, 2024)—and they clearly show that the regulation of sexualized relations at work is an issue that sociological analysis must now address. From this perspective, the concept of a continuum of sexualized relations at work is useful because it allows us to capture, within a single analytical framework, the diversity of situations and regulatory mechanisms in specific workplace contexts, as well as the ambivalent effects—whether protective or potentially reinforcing domination—that they have on individuals and work groups. Finally, its main contribution is that it integrates the three analytical levels—legal, organizational and sociological—while moving beyond their respective binary approaches to sexualized relations in the workplace (lawful/unlawful; benefit/risk; agency/domination).

## Footnotes

1 In this article, we provide English translations of direct quotations from French-language scientific literature.

2 The term encompasses multidisciplinary research fields such as human resources, organizational psychology and behavior, organizational studies or even management (Panda and Puri, 2025). They focus on organizational dynamics and management practices.

3 There is little data and analysis available on cases and dynamics other than heterosexual ones (Williams and Giuffre, 2011).

4 For example, for *Web of Science*, one of the selected queries was: (“work^*^” AND “romance^*^” OR “love^*^” OR “sex^*^” OR “sexuality^*^” OR “sexualization^*^”; Topic). Initially, the OR operator was widely used to account for variation in the formulations and concepts used in the literature. This allowed us to expand the scope of the search and mitigate the risk of excluding relevant studies due to an overly limited vocabulary.

5 Some reviews place academic research on the same level as articles in specialized journals or quantitative data from employment-related institutions (professional associations, media, job platforms). As a result, their findings must be interpreted with caution and critical distance.

6 Research in organizational and management studies mainly relies on quantitative data covering disparate realities (e.g., sexualized relations that have been experienced or witnessed; Anand et al., 2023).

7 From a functionalist and instrumental perspective, the studies highlight the use of sexuality at work as a strategic tool for social influence, enabling individuals to increase their capital within the professional sphere and beyond (Aquino et al., 2014).

8 This does not prevent “legal polysemy” from prevailing (Lamb, 2008, p. 181).
